# A functional assessment of anatomical variants of the recurrent laryngeal nerve during thyroidectomies using neuromonitoring

**DOI:** 10.1007/s12020-017-1466-3

**Published:** 2017-11-08

**Authors:** Beata Wojtczak, Krzysztof Kaliszewski, Krzysztof Sutkowski, Marek Bolanowski, Marcin Barczyński

**Affiliations:** 1 Department and Clinic of General, Gastroenterological and Endocrine Surgery Wroclaw Medical University, Wroclaw, Poland; 2 Department of Endocrinology, Diabetes and Isotope Therapy, Wroclaw Medical University, Wroclaw, Poland; 3 Department of Endocrine Surgery, Third Chair of General Surgery, Jagiellonian University Medical College, Kraków, Poland

**Keywords:** Thyroid surgery, Recurrent laryngeal nerve, Neuromonitoring

## Abstract

**Purpose:**

The aim of the study was to assess the usefulness of intraoperative neuromonitoring (IONM) in identifying anatomical variants of the recurrent laryngeal nerve (RLN) during thyroidectomies, with emphasis on the nerve’s relationship to the inferior thyroid artery (ITA), Zuckerkandl’s tubercle, nonrecurrent laryngeal nerves (NRLNs), and extralaryngeal bifurcation.

**Methods:**

A total of 128 subjects undergoing surgery for thyroid disorders were enrolled in the study, and the course and anatomical variants of RLN were assessed with IONM application.

**Results:**

The standard relationship between RLN and ITA was that the artery and nerve intersect (100%). The right RLN was below the ITA in 76.67% of the patients, and the left RNL was below it in 75.81%. There were no statistically significant differences in the relationship between RLN and ITA on the two sides; and gender did not significantly influence the relationship between the RLN and ITA on either side. In one patient a nonrecurrent inferior laryngeal nerve was present on the right side (0.83%). RLN bifurcation was observed in 33.33% of the patients on the right and in 19.35% on the left side; the difference between sides was statistically significant (*p* < 0.05). Posterior tubercle (Zuckerkandl’s tubercle) was observed on the right in 83% of the subjects and on the left in 69%. The age, thyroid volume and body mass index (BMI) did not influence the size of the tumor.

**Conclusions:**

The utilization of IONM of the RLN in thyroid surgery adds a new dimension to the standard of visual nerve identification allowing for functional nerve testing at the most vulnerable area of the dissection: at the level of Berry’s ligament, posterior tubercle (Zuckerkandl’s tubercle) and crossing of the RLN with the inferior thyroid artery.

## Introduction

Recurrent laryngeal nerve (RLN) palsy is one of the most serious complications after thyroid surgery. The average incidence of temporary postoperative vocal fold palsy is approaching 10% and incidence of permanent RLN injury is 2.3% [1]. The wide range of RLN injury rates found in the literature—from 2.3 to 26%—is related to whether and when a laryngeal examination was performed after surgery, the type of disease (especially the incidence of large goiter), the reoperation rate, the occurrence of massive nodal metastases in compartment VI, the extent of the thyroid operations and the surgeons’ experience [1–3]. Patients with unilateral RLN injury could be asymptomatic or complain of hoarseness and swallowing problems due to incomplete glottic function. Bilateral RLN injuries are uncommon (less than 1% of all RLN injuries), but are always serious complications with respiratory distress, in some cases requiring emergency tracheostomy [4]. In order to prevent RLN injury, routine exposure and preservation of this structure should be standard procedure during all thyroid operations [5–7]. Accurate knowledge of the anatomy of the RLN and its abnormalities, variations and relationships to other tissues is crucial for a safe thyroidectomy, because unexpected anatomical variations of the RLN through its cervical course are among the risk factors for injury to it [8]. In 1938 Lahey was first to show that routine visual RLN identification during thyroid operations reduces the rate of RLN injury, and nowadays there is no doubt that it is the gold standard in thyroidectomy [9]. In 1966 Shedd introduced neuromonitoring in thyroid surgery, which significantly influenced both surgical skills and the rate of RLN injury [10]. In the last two decades intraoperative neuromonitoring (IONM) of the RLN as an adjunct to direct visualization has gained widespread acceptance among surgeons. Moreover, IONM provides information not only about the integrity of the RLN, but also about its functioning, which is a milestone in thyroid surgery [11, 12]. Furthermore, the introduction of IONM has broadened surgeons’ knowledge about the anatomy of the RLN and its variants. These include the variable course of the RLN at the level of the inferior thyroid artery (ITA), the relationship between the RLN and posterior tubercle (Zuckerkandl’s tubercle), nonrecurrent laryngeal nerves (NRLN), and extralaryngeal bifurcation [13].

The RLN was first described by Galen in the second century AD [14]. The nerve consists of motor, sensory and autonomic fibers. It branches from the vagus nerve in the superior part of the thorax, on the left side at the level of the arch of the aorta and on the right at the subclavian right artery. As Randolph wrote, “The RLN becomes recurrent by turning back on itself in the chest emerging superiorly back into the central neck to provide motor innervation to all laryngeal intrinsic muscles except the cricothyroid muscle.” The right RLN extends along the right tracheoesophageal groove, while the left RLN ascends more vertically in the left tracheoesophageal groove [15–17]. On both sides the RLN may cross above or below the ITA, or between its branches [18]. The mean diameter of the RLN ranges from 1 to 3 mm. The left nerve is usually 12 cm between its origin in the vagus nerve and its entrance into the larynx; the right RLN is about 7 cm long [15, 16]. On rare occasions (0.3–0.8%) the right RLN does not recur, and originates from the cervical portion of the vagus nerve. Nonrecurrence of the right inferior laryngeal nerve results from a vascular anomaly during the embryonic development of the aortic arches [19]. About 30–76% of RLNs are branching, usually in the last 2 cm prior to laryngeal entry at the level of Berry’s ligament. Most bifid RLNs have two branches: the anterior (motor branch) and posterior (sensory branch) [20]. The RLN runs close to the posterior capsule of the thyroid before it enters the larynx. This course is sometimes more complicated due to the presence of posterior tubercle, lying very close to the RLN and the superior parathyroid gland. An enlarged posterior tubercle significantly influences the course of the RLN [21].

The aim of this study was to evaluate the usefulness of IONM in identifying anatomical variants of the RLN in thyroid surgery. The study also investigated correlations between the course of the RLN and the patients’ age, sex, body mass index (BMI), thyroid and tumor volume, the side of the thyroid operated on, and the prevalence of posterior tubercle.

## Material and methods

The study was approved by the Bioethics Committee of Wroclaw Medical University (Wroclaw, Poland). Between January 2012 and December 2014, 236 thyroid operations were performed with IONM at the Department of General, Gastroenterological, and Endocrine Surgery at Wroclaw Medical University by three surgeons. Out of this group, 128 patients (114 women and 14 men) operated by one surgeon with detailed assessments of the course of the RLN were enrolled in the study. The mean age of the study group was 55 ± 16 years (ranging from 17 to 83). Thyroid reoperations and suspicion of anaplastic cancer were exclusion criteria for the study because of the extremely difficult operating field in such cases. The participants had undergone primary thyroid operations due to multinodular goiter (103 patients), Graves’ disease (8 patients) and papillary cancer (17 patients); 116 of the patients underwent thyroidectomy and 12 lobectomy. A total of 244 RLNs at risk were identified with the use of IONM: 120 on the right side of the thyroid and 124 on the left side.

Before surgery all the patients were euthyroid; both thyrotropin (TSH) and free thyroxine (fT4) levels were measured before the operations. Among the patients with multinodular goiter, 35% were treated due to hyperthyroidism to prevent thyroid storm. The hyperthyroid patients were treated with antithyroid drugs: thionamide and beta-blocking agents or by 131I.

The Lugol’s solution was not used in the period before the operation. Along with the improvement of the operating technique (bloodless field) the Lugol’s solution is no longer used in our Department. Ultrasonographic examinations of the thyroid and chest X-rays were performed to assess the volume of the thyroid and any potential dislocation and compression of the trachea.

Intermittent neuromonitoring with NIM-3.0 equipment (Medtronic, Jacksonville, FL, USA), was used during all the operations. A monopolar stimulating probe with a handle was used for nerve stimulation with a current amplitude of 1 mA to 2 mA and 3 Hz impulses of 200 ms each for 1–2 s. The electromyographic (EMG) signal was obtained using surface electrodes integrated in the endotracheal tube (NIM Flex EMG tube, Medtronic); in female patients 7 mm tubes were used, and in males 7.5–8 mm tubes were used. Monitoring of the RLN was done in accordance with the standards proposed by International Neuromonitoring Study Group (L1, V1, R1, R2, V2, and L2). All the patients underwent laryngeal examinations before their thyroid operations (L1). Before the dissection of the lobe of the thyroid, the vagal nerve was stimulated (V1) first, then the recurrent laryngeal nerve before (R1) and after (R2) removing the lobe. At the end of the operation the response of the vagal nerve to stimulation was assessed to confirm its functional integrity (V2) [11]. Postoperative laryngoscopy was routinely used to diagnose any RLN injury on the first or 2nd day after the operation (L2). RLN stimulation at 1 mA was used to confirm the course of the nerve, and 2 mA was used for mapping. In cases where similar structures close to each other needed to be distinguished, 0.5 mA stimulation was used. The non-recurrence nerve was identify with utilization of neuromonitoring based on short latency below 3.5 ms and lacking EMG response after stimulation from the vagus nerve at the bottom of the neck.

Anatomical variants of the RLN and the prevalence of posterior tubercle (Zuckerkandl’s tubercle) influencing the course of the nerve were assessed during the thyroid operations. In particular, relationships between the RLN and ITA, the prevalence of non-RLNs and RLN branching was investigated. Correlations between anatomical variants of the RLN and the patient’s age, sex, BMI or the side of the thyroid lobe were investigated. The size of posterior tubercle was evaluated using the Pelizzo grading system: a numerical scale from 0 to 3, with 0 being unrecognizable, 1 being only a thickening of the lateral edge of the thyroid lobe, 2 measuring smaller than 1 cm, and 3 measuring larger than 1 cm in size [22]. Correlations between the prevalence of this tubercle and the patient’s sex, age and the volume of the thyroid were investigated.

To assess anatomical variants of the RLN, different approaches to the nerve were used. Lateral and inferior approaches were the most common in routine thyroid operations. The superior approach was useful in cases of large cervical goiters or substernal localization.

The RLN variants and posterior tubercle were documented in a database as well as by video and photographically. A statistical analysis was carried out using Prism 5.0 statistical software (Graphpad, La Jolla, CA, USA). Relationships among the clinicopathological parameters were analyzed by Fisher’s exact test and the *χ* ^*2*^ test. To compare parametric data, the unpaired Student’s test was used, while the Mann–Whitney U test was used to compare groups of data that did not meet the assumptions of the parametric test. To compare more than two groups, the Kruskal–Wallis test was applied, with a post hoc analysis using Dunn’s multiple comparison test.

## Results

### RLN and ITA intersection

The standard relationship between the RLN and ITA is that the artery and nerve intersect (100%) (Table 1). The most common course of the RLN was under the ITA on both sides: 76.67% on the right and 75.81% on the left. The RLN running superficially to the ITA was observed less than in one in four cases: 19.17% on the right side and 24.19% on the left. An RLN between the branches of the ITA was observed in only 3.33% of the cases, on the right side. There were no statistically significant differences between the two sides in terms of the relationship between the RLN and ITA (*p* > 0.05). The patient’s sex had no significant influence on the relationship between the RLN and ITA on either side (*p* > 0.05).

**Table 1:** The relationship between the recurrent laryngeal nerve and inferior thyroid artery

Relationship of RLN and ITA (244 RLNs)	RLN posterior to the ITA	RLN anterior to the ITA	RLN between branches of the ITA	*χ* ^*2*^test
On the right side (120 RLNs)^a^	92 (76.67%)	23 (19.17%)	4 (3.33%)	*p* = 0.1496
On the left side (124 RLNs)	94 (75.81%)	30 (24.19%)	–	
Women (*n* = 114) (216 RLNs)^a^	166 (76.49%)	46 (21.2)	4 (1.84%)	*p* = 0.72897
Men (*n* = 14) (27 RLNs)	20 (74.07%)	7 (25.92%)	–	

*RLN* recurrent laryngeal nerve, *ITA* inferior thyroid artery, *ns* not significant

^a^ One nerve on the right side was a nonrecurrent inferior laryngeal nerve

### The nonrecurrent inferior laryngeal nerve

In one patient a nonrecurrent inferior laryngeal nerve was present on the right side (0.83%). The NRLN was found in a 34-year-old woman undergoing surgery for a multinodular goiter. On the left side of the thyroid the RLN was found under the ITA.

### Recurrent laryngeal nerve branching

RLN bifurcation was observed in 33.33% on the right side and in 19.35% on the left side; this difference was statistically significant (*p* < 0.05) (Table 2). On both sides, the most common bifurcation observed was the RLN subdividing into two branches (70% of the bifurcations on the right and 67% on the left) but as many as five branches were noted. In 17 patients (13.28%) the RLN bifurcated on both the right and left sides. On the right side the RLN often bifurcated less than 2 cm before the laryngeal entry point; on the left side 58.33% of the bifurcations were observed at a more proximal point (more than 2 cm from the larynx), but the differences were not statistically significant. There were no statistically significant differences between the two sexes in terms of the number and location of the bifurcations (*p* > 0.05). Usually the anterior branch was larger than the inferior one, but the diameter of the branches was not measured.

**Table 2:** Bifurcations of the recurrent laryngeal nerves

	RLN on the right side (*n* = 120)	RLN on the left side (*n* = 124)	*χ* ^2^ test	Women (*n* = 114, 217 RLN)	Men (*n* = 14, 27 RLN)	*χ* ^2^ test
Non-bifurcating RLN	80 (66.67%)	100 (80.65%)	*p* = 0.0159*	161 (75%)	17 (63%)	*p* = 0.1856
RLN branching (*n* = 100%)	40 (33.33%)	24 (19.35%)		54 (25%)	10 (37%)	
Bifurcation of the RLN into two branches	28 (70%)	16 (67%)	*p* = 0.7800	39 (72%)	5 (50%)	*p* = 1.637
Bifurcation of the RLN into more than two branches	12 (30%)	8 (33%)		15 (28%)	5 (50%)	
Bifurcation <2 cm from the larynx	16 (67%)	10 (41.67%)	*p* = 0.8002	21 (40%)	4 (40%)	*p* = 0.9747
Bifurcation >2 cm from the larynx	10 (33%)	14 (58.33%)		32 (60%)	6 (60%)	

*RLN* recurrent laryngeal nerve

The threshold of statistical significance (*) was p < 0.05

### The recurrent laryngeal nerve and posterior tubercle (Zuckerkandl’s tubercle)

In all the patients the RLN was behind posterior tubercle, regardless of which side it was on. The tubercles were more often observed on the right side (83%) than on the left (69%). The size of the tubercles was assessed according to Pelizzo grading (Table 3); they were smaller than 1 cm in 42% of the cases on the right side and in 27% of the cases on the left side. There were statistically significant differences between the two sides of the thyroid in terms of the size of the tubercles; on the right side the tubercles were generally larger than on the left (*p* < 0.05). There were no correlations between compression and dislocation of the trachea shown in X-ray chest examinations and the size of posterior tubercle (*p* > 0.05). There was, however, a statistically significant correlation between the size of posterior tubercle and the presence of retrosternal goiter (*p* < 0.05).

**Table 3:** Grading of Zuckerkandl’s tubercle

Zuckerkandl’s tubercle according to Pelizzo grading	0 unrecognizable	1 thickening only	2 smaller than 1 cm	3 larger than 1 cm	*χ* ^2^ test
Right lobe of the thyroid	21 (18%)	12 (10%)	50 (42%)	37 (31%)	*p* = 0.00282
Left lobe of the thyroid	39 (31%)	24 (19%)	33 (27%)	28 (23%)	
women (*n* = 114, 217 RLN)	52 (23.96%)	32 (14.75%)	78 (35.94%)	55 (25.35%)	*p* = 0.29703
men (*n* = 14, 27 RLN)	8 (29.63%)	4 (14.81%)	5 (18.53%)	10 (37.03%)	
normal chest X-ray (*n* = 138)	29 (21.01%)	19 (13.77%)	53 (38.41%)	29 (26.81%)	*p* = 0.9863
compression, dislocation of the trachea in chest X-ray (*n* = 58)	31 (53.45%)	17 (29.31%)	30 (51.72%)	36 (34.48%)	
cervical goiter (*n* = 186)	41 (22%)	31(16%)	74 (39%)	43 (23%)	*p* = 0.0133
retrosternal goiter (*n* = 54)	19 (35%)	5 (9%)	9 (16%)	22 (40%)	

The size of posterior tubercle correlate with the age of the patient, thyroid volume and BMI. The patient’s age had no influence on the size of the tumor (*p* = 0.319), nor did BMI (*p* = 0.2813). The volume of the thyroid in ultrasonography did not correlate with the size of posterior tubercle on either side (Table 4).

**Table 4:** Zuckerkandl’s tubercle and mean age, BMI, thyroid volume

Zuckerkandl’s tubercle	Mean age	BMI	Thyroid volume/ultrasound (ml)
Unrecognizable	49.82	24.76	37.73
Thickening only	53.90	25.91	25.86
Smaller than 1 cm	57.22	27.97	38.29
Larger than 1 cm	53.57	26.64	53.09
All groups	54.50	26.84	43.26
Kruskall-Wallis test	*p* = 0.419	*p* = 0.2812	*p* = 0.1559

## Discussion

The relationship between the attempts to preserve the RLN and the maintenance of the patient’s normal voice after thyroid operations has been known for a long time and has been confirmed by Lahey and Halstead in 20th century [9, 23]. Nowadays there is no doubt that knowledge of anatomy and surgical skills in identification of the RLN is basic to modern thyroid surgery [2, 5, 7]. The RLN and its anatomical variants can be identified by visual inspection alone, but use of neuromonitoring may help not only in the anatomical nerve identification but also in the functional preservation of the nerve. In previous studies the authors showed that the use of IONM improved accuracy of the RLN identification among low to medium volume thyroid surgeons, while it was of a limited added value for expert hands of high volume surgeons [24, 25]. Moreover visual RLN identification alone may be unreliable in some cases; in RLN bifurcations and in non-recurrent RLN or could be not sufficient in distinguishing between the branches of the RLN and small branches of ITA or with any others structures in the neck which mimic the nerve. That’s why the RLN’s evaluation with utilization of neuromonitoring gives the surgeons a new dimension of functional feedback for more accurate nerve identification. In addition, despite the fact that visual RLN identification still remains a gold standard in thyroid surgery, the IONM has challenged the paradigm of anatomical nerve identification by giving an adjunct for functional nerve testing which in many clinical situations may be of additional value in surgical decision-making [11–13].

Knowledge of the possible relationships between the RLN and the ITA is important, because the artery is often used as a landmark for the position the nerve. Mistaking a branch of the ITA for the RLN can lead to a transection injury [5]. The basic relationship is that the RLN and the main trunk or branches of the ITA intersect, which has been confirmed in the present study (100%). The relationships can, however, vary greatly—Reed described 28 different ones [26]—but generally two main relationships are observed: The RLN is either deep or superficial to the ITA. In the present study the RLN was deep to the ITA in 76.67% of the cases on the right side and in 75.81% of the cases on the left. A higher incidence of the RLN running posterior to the ITA was observed in a review of 17 studies from a period of 70 years [18]. In a study by Hollingshead, in about 50% of the cases the RLN was deep to the ITA; in 25% it was superficial and in 25% it was between branches of the ITA [27]. In the present study there were no differences between the two sides of the thyroid in terms of in the course of the RLN relative to the ITA, but in a study by Campos statistically significant differences were found between in the two sides in terms of the relationship of the RLN to the ITA. In his study the nerve most often intertwined with branches of the ITA on both sides; on the right side the RLN was anterior to the ITA in 38.04% of the cases and posterior to it in 11.26%; on the left side, it was posterior to the ITA in 37.05% of the cases and anterior to it in 18.05% [18]. Usually the relationships found on one side did not occur on the opposite side; in Campos’s study the course of the RLN relative to the ITA differed on the two sides in 62.68% of the cases [18].

Some publications have assessed the course of the RLN relative to the ITA according to sex. For example, Lee et al. reported that the nerve was posterior the ITA in females more often than in males (51.7 vs. 38.8%) [28]. The present study found no correlation between the patient’s sex and the relationship between the RLN and ITA on either sides (*p* > 0.05).

One possible, but very rare, anatomic variation of the RLN is the nonrecurrent inferior laryngeal nerve. In the present study it was observed only in one patient, on the right side (0.83%). The use of IONM with the recommended algorithm is helpful both in identifying this variant and in protecting it from inadvertent injury. The absence of a response to distal vagus stimulation suggests a potential NRLN, and positive proximal vagal stimulation confirms the presence of an intact NRLN [11]. The available literature indicates that the prevalence of a right-side NRLN is 0.6–1.0%; left-side NRLNs are extremely rare, with only a few reported cases in the literature [29].

The next point of consideration in the present study was the prevalence of recurrent laryngeal nerve branching. According to various surgical studies, the RLN branches before entering the larynx in 30–78% of the cases [16]. In this study RLN bifurcations were less frequent than in the literature: in 33.33% of the RLNs on the right side and in 19.35% on the left side; the difference was statistically significant (*p* < 0.05). In our experience the small, thin extralaryngeal branches can be almost invisible, which may contribute to the wide variations in the frequency of RLN branching reported in the literature. The RLN usually subdivides into two main branches: the anterior and posterior. In the present study this type of bifurcation was the most common on both sides: 70% on the right and 67% on the left. The branching of the nerve is usually in the distal RLN segment, in the last 2 cm, and this part of the nerve is at an extremely high risk of injury [30]. RLN branching point distances (the distance from the point of bifurcation to the RLN’s entry into the larynx) were measured by Serpell et al. who found that the median branching distance was 18 mm on the right and 13 mm on the left, with a range of 5–34 mm [31, 32]. The present study confirmed those observations on the right side, where the RLN branching point distance was less than 2 cm in most cases (67%), but in contrast to that study, on the left side the branching point distance was more than 2 cm in most cases (67%). When the RLN is bifurcated, the branching usually occurs close to Berry’s ligament; in rare cases the branching is below the ITA [32, 33]. In the present study bilateral branching was observed in 13.28% of the patients. Other authors have reported bilateral branching in as many as 28.6% of the patients [30, 32–34]

The present study investigated relationships between RLN branching (prevalence, type and the distance from the larynx) with the patient’s sex, but no statistically significant differences were found in the study group. In contrast, in a big retrospective study on 491 patients, Fontenot et al. observed racial and gender variations in the prevalence of RLN branching. Their study showed that African-American patients had a higher rate of bifurcation (42.1%) compared with Caucasians (33.2%). Moreover, among females bifurcations were observed at longer distances from the larynx than in men [20].

According to the present study as well as the reports of other authors, the right side is at particularly high risk of injury during thyroidectomies due to the higher incidence of bifurcations. Regarding RLN bifurcation, it is important to highlight the role of IONM in assessing the functional status of the nerve. IONM allows surgeons to distinguish branches of the RLN from other structures, and to differentiate between sensory and motor branches [7, 11, 13].

The final point of the present study waks the assessment of posterior tubercle and its relationships to the RLN. Posterior tubercle is of great interest to thyroid surgeons because of its close proximity to the RLN and parathyroid glands. In the present authors’ experience, in primary operations the nerve always runs under the tubercle. We have observed RLNs overlapping the tubercle in cases of recurrent goiter, but reoperations were excluded from the study. Most research on posterior tubercle has found a higher incidence on the right side [22, 35, 36]. This was confirmed in the present study, in which the rate of posterior tubercle was 83% on the right side and 69% on the left side (*p* < 0.05). The size of the tubercle was higher on right side as well (*p* < 0.05). We tried to predict the presence of posterior tubercle based on X-rays, but there were no correlations with tracheal compression or dislocation. Likewise, neither thyroid volume, the patient’s sex, age nor BMI had any influence on the prevalence of the tubercle. There was, however, a statistically significant correlation between the size of posterior tubercle and the presence of retrosternal goiter (*p* < 0.05).

This study has several limitations. First of all the study group comprised of 128 patients and hence it can be considered a relatively small study. On the other hand, all the data were collected prospectively and maintained in a computerized database, which ensures their high credibility. Second, all these anatomical variants were observed by a single high volume thyroid surgeon which may be difficult to follow by low volume thyroid surgeons.
